# Anti-PD1 does not improve pyroptosis induced by γδ T cells but promotes tumor regression in a pleural mesothelioma mouse model

**DOI:** 10.3389/fimmu.2023.1282710

**Published:** 2023-11-23

**Authors:** Ka Sin Lui, Zuodong Ye, Hoi Ching Chan, Yoshimasa Tanaka, Allen Ka Loon Cheung

**Affiliations:** ^1^ Department of Biology, Faculty of Science, Hong Kong Baptist University, Hong Kong, Hong Kong, SAR, China; ^2^ Center for Medical Innovation, Nagasaki University, Nagasaki, Japan

**Keywords:** gamma-delta T cells, mesothelioma, pyroptosis, anti-PD1, pleural mesothelioma

## Abstract

**Introduction:**

Mesothelioma is an aggressive tumor in the pleural cavity that is difficult to treat. Diagnosis is usually late with minimal treatment options available for the patients and with unfavorable outcomes. However, recent advances in immunotherapy using γδ T cells may have potential against mesothelioma, given its ample tumoricidal and tumor-migratory properties could allow its infiltration to the widespread tumor mass. Thus, we hypothesize that Vδ2 T cells can perform cytotoxic activities against mesothelioma especially when combined with immune checkpoint blocker against PD-1.

**Methods:**

Human Vδ2 T cells were expanded from peripheral blood mononuclear cells using Tetrakis‐pivaloyloxymethyl 2‐(thiazole‐2‐ylamino) ethylidene‐1,1‐bisphosphonate (PTA) plus IL-2 for 13 days, before used to test for cytotoxicity against mesothelioma cell lines. Mesothelioma-bearing mice was established by Intrapleural administration of mesothelioma cell lines to test for the efficacy of Vδ2 T cells plus anti-PD-1 antibody combination treatment. Pyroptosis was evaluated by cell morphology, western blot analysis, and ELISA experiments. Flow cytometry was used to examine expression of BTN2A1, BTN3A1, PD-L1, PD-L2 on mesothelioma cell lines. Immunofluorescence staining was performed to detect Vδ2 T cells post adoptive transfer and characteristics of pyroptosis in *ex vivo* mesothelioma tissue sections.

**Results:**

Indeed, our data demonstrated that Vδ2 T cells killing mesothelioma can be enhanced by anti-PD-1 antibody *in vitro*, especially for high PD-1 expressing cells, and in vivo in the intrapleural mesothelioma mice model established by us. Adoptive transfer of Vδ2 T cells into these mice leads to tumor regression by 30-40% compared to control. Immunofluorescence of the tumor section confirmed infiltration of Vδ2 T cells into the tumor, especially to cells with BTN2A1 expression (a Vδ2 T cell activating molecule) despite PD-L1 co-localization. Interestingly, these cells co-expressed cleaved gasdermin D, suggesting that pyroptosis was induced by Vδ2 T cells. This was verified by Vδ2 T/mesothelioma co-culture experiments demonstrating membrane ballooning morphology, increased cleaved caspase-3 and gasdermin E, and upregulated IL-1β and IL-18.

**Discussion:**

Vδ2 T cells plus anti-PD1 exhibited cytotoxicity against mesothelioma in vivo. However, we found no advantage for anti-PD-1 against PD-1 high expressing Vδ2 T cells in promoting pyroptosis. Taken together, our work demonstrated that Vδ2 T cells combined with anti-PD-1 antibody can be developed as a potential combination immunotherapy for mesothelioma.

## Introduction

Mesothelioma is an aggressive cancer that occurs in the mesothelial lining of the pleura, pericardium, and peritoneum (1). It is primarily caused by exposure to asbestos in construction materials during the 1950s, leading to the transformation of mesothelium cells into tumor cells with a latency period of over 30 years (1–3). In 2020, at least 26,000 mesothelioma-related deaths were reported globally (4), and the incidence and mortality rates are expected to rise, especially in undeveloped countries or cities where asbestos is still used. Unfortunately, the survival rate for mesothelioma patients remains low, and the available treatment options, including chemotherapy, surgical resection, and certain immunotherapies, only provide limited improvements in patient lifespan (5). Moreover, traditional therapies often result in unsatisfactory outcomes, with serious complications such as empyema leading to death (6, 7). Additionally, the lack of accurate and reliable biomarkers for mesothelioma detection hinders the widespread use of advanced immunotherapies like CAR CD8^+^ T cells, which require high antigen specificity (8). In light of these challenges, alternative natural immune cells with tumoricidal properties that do not rely on antigen recognition may hold potential against mesothelioma.

Pyroptosis is a form of programmed cell death mediated by inflammatory caspases like Caspase 1, 3, 4, 5, 11, which enables an inflammatory response and the release of active IL-1β and IL-18 (7, 9). Pyroptosis is characterized by membrane blebbing, where cleaved gasdermin D and E proteins form membrane pores that induce cell swelling, rupture, and the release of cytokines (10–12) The cleavage of gasdermin D is mediated by the non-canonical activation of caspase 4, 5 or 11, while gasdermin E is cleaved by canonical pathway activated caspase 1 or 3 (10, 12–16). Pyroptotic cell death activates anti-tumor immune responses, making it a focus of cancer treatment research. Recent studies have shown upregulated pyroptosis-related genes in mesothelioma compared to other cancers, correlating with the susceptibility of mesothelioma cells to pyroptosis induction (15, 17). Therefore, utilizing Vδ2 T cells in our model may hold therapeutic potential for inducing pyroptosis and improving clinical outcomes for mesothelioma patients.

The Vδ2 subset of gamma-delta T cells (γδ T cells) possesses ample cytotoxicity against various cancers, including cholangiocarcinoma, pancreatic cancer, and lung cancer (18–20). We recently shown in a nasopharyngeal carcinoma mice model that Vδ2 T cells can infiltrate into tumor mass, particularly to areas of cells that express BTN2A1/BTN3A1 (21). These molecules facilitate the presentation of phosphoantigen (pAg) and subsequent activation of Vδ2 T cells, with increased pAg found in tumor cells. Importantly, Vδ2 T cells can be robustly expanded *in vitro* using a prodrug called tetrakis-pivaloxloxymethyl 2-(thiazole-2-ylamino) ethylidene-1,1-bisphosphonate (PTA), which exhibits cytotoxicity against mesothelioma through three distinct mechanisms (21–23).

Despite the advantages of Vδ2 T cells, they can become “exhausted” in the tumor microenvironment due to immune checkpoint molecules such as PD-1 and the ligands PD-L1 and PD-L2. Immune checkpoint inhibitors like nivolumab (anti-PD-1 antibody) or durvalumab (anti-PD-L1 antibody) are used clinically to restore the cytotoxic function of immune cells by blocking the PD-1/PD-L1 interaction (9, 24). These inhibitors have shown to extend the lifespan of mesothelioma patients by at least 10 months (25–30). Thus, we hypothesized that combining anti-PD-1 antibody with Vδ2 T cells can enhance the efficacy against mesothelioma.

Our data shows that anti-PD-1 antibody (nivolumab) can enhance the anti-tumor ability of Vδ2 T cells against mesothelioma *in vitro* in a pleural mesothelioma mice model, especially those cells with high PD-1 expression. Immunofluorescence staining of tissue sections revealed an increased number of tumor infiltrating Vδ2 T cells. Interestingly, live-imaging of Vδ2 T cells co-cultured with mesothelioma showed the induction of pyroptosis in the cells, which was confirmed by the detection of the active Caspase 3 and gasdermin E protein expressoion, as well as increased IL-1β and IL-18. However, the pyroptotic effect was not enhanced by anti-PD-1 antibody.

## Materials and methods

### Cell lines

Human mesothelioma cell lines MSTO-211H (hereafter referred to as MSTO) and NCI-H2052 cells (hereafter referred to as H2052) were purchased from ATCC, were cultured with RPMI 1640 medium (ATCC modification) (Cat. no. A1049101, GIBCO) supplemented with 10% fetal bovine serum (FBS) (Cat. no. 10270106, GIBCO). Cells were incubated at 37°C in 5% CO_2_. MSTO and H2052 cells are derived from the lung of male patients who suffered from biphasic and stage 4 mesothelioma, respectively.

### Construction of luciferase reporter mesothelioma cell lines

pLV-Fluc-mCherry-Puro plasmid encoding luciferase reporter gene and mCherry gene (provided by Yue Jianbo, City University of Hong Kong) and the two packaging plasmids, pMD2.G (Cat. no. 12259, Addgene) and psPAX2 (Cat. no. 12260, Addgene) were co-transfected into 293T cells to generate lentiviruses. After 48 h post-transfection, the supernatant containing lentiviruses was collected and used to transduce the luciferase gene into MSTO or H2052 cells. Polybrene (10 μg/ml) was added to the cells to improve the lentiviral infection efficiency. After overnight incubation, MSTO or H2052 cells that tested positive for mCherry were selected by replacing culture medium supplemented with puromycin (2 μg/ml) after 2 days post-infection. Single-cell clones were further obtained by limiting dilution methods, and the luciferase activity was verified by Perkin Elmer EnSight Microplate Reader. The stable luciferase reporter mesothelioma cell lines – MSTO-luc and H2052-luc were then established.

### Expansion and purification of γδ T cells *in vitro*

Peripheral blood mononuclear cells (PBMC) were isolated from human whole blood (Hong Kong Red Cross) using density gradient medium (Lymphoprep, Cat. no. 07861, STEMCELL Technologies). Tetrakis‐pivaloyloxymethyl 2‐(thiazole‐2‐ylamino) ethylidene‐1,1‐bisphosphonate (PTA) (PTA; 1 μM/ml; kindly provided by Prof Yoshimasa Tanaka, Nagasaki University) were used to stimulate the expansion of PBMC (4 x 10^6^ cell/ml) in recombinant human IL-2 protein (rhIL-2; 100 IU/ml; Cat. no. 202-IL-500, R&D systems) containing RPMI 1640 medium (Gibco) supplemented with 10% FBS (GIBCO) at 37°C in 5% CO_2_ incubator for 13 days with 50% media change every 2-3 days. After 13 days in culture, γδ T cells were purified by human TCR γ/δ^+^ T Cell Isolation Kit (Cat. no. 130-092-892, Miltenyi Biotec). The purity of CD3^+^Vδ2^+^ cells were achieved to >95% before used for subsequent experiments.

### Western blotting

MSTO-luc and H2052-luc were co-cultured with pre-treated γδ T cells using nivolumab (anti-PD-1 antibody) (Cat. no. HY-P9903, MedChemExpress) for 6 hours. 10 μM raptinal (Cat. no. HY-121320, MedChemExpress) and 20 μM terfenadine (Cat. no. HY-B1193, MedChemExpress) served as positive controls, mesothelioma cells alone served as negative control. Protein extraction from the cells was performed using denaturing lysis buffer (31). Protein concentrations were measured using Pierce BCA Protein Assay Kit (Cat. no. 23227, Thermo Scientific), 20 μg protein lysates were loaded into 10% SDS-PAGE gel for electrophoresis, followed by wet-transfer onto Immobilon PVDF membrane (Cat. no. ISEQ00005, Sigma-Aldrich) in Trans-Blot Electrophoretic Transfer Cell (Bio-Rad). Membranes were blotted with 5% skimmed milk (Blotting-grade Blocker, Cat. no. 1706404, Biorad) and 0.5% BSA (Cat. no. A3983, Sigma-Aldrich) in TBS-T (1x Tris-buffered saline with 0.1% Tween-20) at room temperature for 1 h. Unconjugated primary antibodies and HRP conjugated secondary antibodies used are shown in **Supplementary Table 1**. The protein bands were detected by ChemiDoc (Bio-Rad) using SuperSignal™ West Pico PLUS Chemiluminescent Substrate (Cat. no. 34580, Thermo Scientific). ImageJ software (http://imagej.nih.gov/ij/) was used to analyze the band intensities.

### Flow cytometry

Cells were collected and washed with FACS buffer (1% FBS in PBS). The cells were then incubated with the appropriate conjugated antibodies, as indicated in **Supplementary Table 1** in 100 μl FACS buffer at 4°C for 30 min for surface protein staining. Cells were analyzed using BD FACSCanto™ II or BD FACSymphony™ A1 flow cytometer.

### Cytotoxicity assay

Mesothelioma cells were pre-labelled with 2 μM Calcein AM (Cat. No. C3100MP, Invitrogen). Luciferase reporter mesothelioma cells were co-cultured with Vδ2 T cells with or without pre-treatment of nivolumab (Anti-PD-1 antibody). Different ratios between Vδ2 T (effector): mesothelioma cells (target) (E:T) were performed in U-round bottom 96 well plate (Cat. No. 168136, Thermo Fischer Scientific) at 37°C in 5% CO_2_ incubator for 4 h. 1% Triton X-100 was used as the positive control to induce maximum cell death. After centrifuging the plate at 400 xg for 5 minutes, the supernatant was transferred to a black 96-well plate, followed by measuring the released Calcein fluorescence signal at excitation wavelengths 495 nm and emission 515 nm wavelengths. Cell lysis percentage was calculated by $\frac{\left(\text{calcein of sample release}-\text{spontaneous release}\right)}{\left(\text{maximum release}-\text{spontaneous release}\right)}\times 100\%$ . Maximum release refers to the positive control. Spontaneous release refers to Calcein-labelled target cells only.

### Live cell imaging using Incucyte S3

To monitor total live and dead mesothelioma cells, mesothelioma cells were pre-labelled with 2 μM Calcein AM. Reporter mesothelioma cells were co-cultured with Vδ2 T cells with or without nivolumab pre-treatment at different E:T ratios in a 96-well flat bottom culture plate (Cat. No. CS016 – 0096, ExCell Bio) and incubated at 37°C in 5% CO_2_ incubator. To track cell death, 250 nM Cytotox Red Reagent (Cat. No. 4632, Sartorius) was added to the culture medium, and the cells were monitored over a 4-hour period. Images were captured at regular intervals of 20-30 minutes using the IncuCyte S3 Live-Cell Analysis System (Sartorius) and analyzed using the Incucyte software (Sartorius).

### Enzyme-linked immunosorbent assay

Different ratios of Vδ2 T cells to MSTO-luc and H2052-luc cells were co-cultured. Vδ2 T cells were pre-treated (or not) with nivolumab for 6 hours in a 96-well plate at various ratios. Controls include tumor cells only, raptinal or terfenadine treatment. After co-culture, the plate was centrifuge at 400 xg for 5 min before collecting the supernatants. Supernatants were stored at -80°C until they were used for the IL-1β ELISA kit, following the manufacturer’s instructions (Cat. No. HSLB00D, R&D Systems). Optical density was measured at 450 nm with the wavelength correction at 540 nm using the microplate reader (BioTek Absorbance Microplate Reader), and the concentration of IL-1β in the samples was calculated.

### Mice experiments

NOD.Cg-*Prkdc^scid^Il2rg^tm1Wjl^*/SzJ (NSG) mice were obtained from the City University of Hong Kong Laboratory Animal Research Unit. All animal experiment were approved by Hong Kong Baptist University Research Ethics Committee (#REC/20-21/0217 and #REC/21-22/0217, #REC/22-23/0438). To establish the xenograft pleural mesothelioma-bearing mice, 5 x 10^6^ MSTO-luc or H2052-luc were injected into NSG mice intrapleurally (i.pl). On day 2, when luciferase signals became detectable, 1 x 10^7^ Vδ2 T cells were adoptive transferred into the mice intravenously (i.v.) in 100 μl PBS. Nivolumab was administered intraperitoneally (i.p.) on day 3 and 6 post-tumor injection. PBS injection served as control. Mesothelioma luciferase activities were measured every 3-4 days by NightOWL II LB 983 *In Vivo* Imaging System (Berthold Technologies).

### Immunofluorescence staining of tumor tissue sections

Tumor were fixed with 10% formalin solution (Cat. no. HT501128, Sigma-Aldrich) followed by dehydration of a series of 70%, 80%, 95%, 100% ethanol and xylene (Cat. no. 1330-20-7, RCI labscan), as well as paraffin (Cat. no. P3808, Sigma-Aldrich) for embedding. 5 μm tumor sections made using the microtome (Shandon Finesse 325 Rotary Microtome, Thermo Fisher Scientific), placed onto adhesive microscope glass slides (Cat. No. 0810501, Marienfeld) and kept at room temperature and in the dark until used. Tumor tissue sections were dewaxed, and antigen retrieval was performed using citrate-based antigen unmasking solution (Cat. No. H-3300, Vector Laboratories). Following blocking with 10% normal goat serum, the tumor sections were stained with unconjugated primary antibodies and conjugated secondary antibodies shown as **Supplementary Table 1**. Images were acquired by Stellaris confocal microscope (Leica). Counting of nucleated cells was based on Hoechst 33258 staining, with combinations three independent experiments.

### Statistical analysis

Statistical analyses were performed using Student’s *t*-test, one-way or two-way analysis of variance (ANOVA), unless otherwise indicated. *P*< 0.05 is considered statistically significant.

## Results

### Human Vδ2 T cells exerts cytotoxicity against mesothelioma cell lines

**Figure 1:**
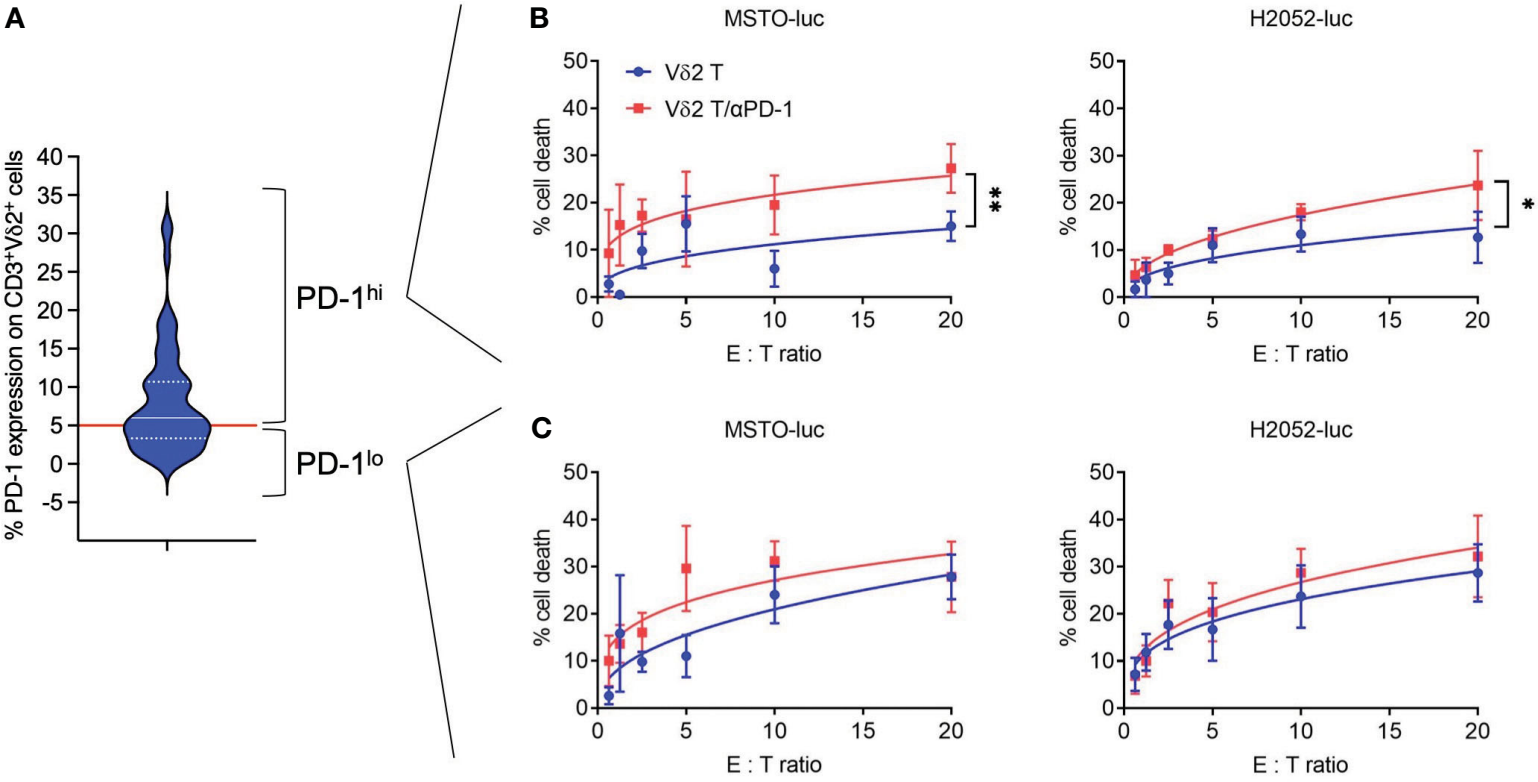
Anti-PD-1 enhances PD-1^hi^ Vδ2 T cells cytotoxicity towards mesothelioma cell lines. **(A)** PD-1 expression on CD3^+^Vδ2^+^ cells analyzed by flow cytometry shown as a violin plot. Red line indicates a cut-off of 5.99% as the median. **(B)** PD-1^hi^ Vδ2 T cells and **(C)** PD-1^lo^ Vδ2 T cells were co-cultured with luciferase reporter-transduced mesothelioma cell lines (MSTO-luc and H2052-luc) at different effector: target (E:T) ratios in a cytotoxicity assay. Data represents mean ± SEM from ≥ 3 independent experiments. Student’s *t*-test was performed. *P< 0.05, **P< 0.01.

Freshly isolated human PBMCs were used to expand Vδ2 T cells for 13 days using PTA and IL-2 following the previously described protocol (21), where cell clusters are formed and expanded (**Supplementary Figure 1A**), with cell numbers that can be increased by 100-1000 fold (**Supplementary Figure 1B**). The purity of the CD3^+^Vδ2^+^ cells reached approximately 80% after expansion and was further enriched to >95% using microbeads (**Supplementary Figures 1C–F**). These cells were then used for cytotoxicity assays against two human mesothelioma cell lines with transduced luciferase expression (MSTO-luc and H2052-luc) (**Supplementary Figure 2**). Considering that PD-1 serves as an indicator of “exhausted” function in Vδ2 T cells, we first analyzed its expression by flow cytometry (**Figure 1A**). The median expression of PD-1 was found to be ~5.99%, which was used as the cut-off to distinguish PD-1^lo^ and PD-1^hi^ Vδ2 T cells. As shown in (**Figures 1B, C**), PD-1^lo^ Vδ2 T cells exhibited cytotoxic responses up to ~25% against MSTO-luc and H2052-luc. In contrast, PD-1^hi^ Vδ2 T cells resulted in ~12% and ~10% of cytotoxicity against MSTO-luc and H2052-luc, respectively. Thus, we tested the effect of pre-treating the cells with αPD-1 (nivolumab) to improve the cytotoxic functions. Indeed, αPD-1 could boost the cytotoxic effect of PD-1^hi^ Vδ2 T cells against MSTO-luc and H2052-luc by ~2-fold significantly (**Figure 1B**). However, the antibody only modestly enhanced cytotoxicity without statistical significance (**Figure 1C**). Therefore, determining the level of PD-1 expression on Vδ2 T cells is crucial in justifying the use of anti-PD-1 immune checkpoint inhibitors as immunotherapy for mesothelioma.

### Vδ2 T cells plus αPD-1 retarded mesothelioma tumor growth *in vivo*

**Figure 2:**
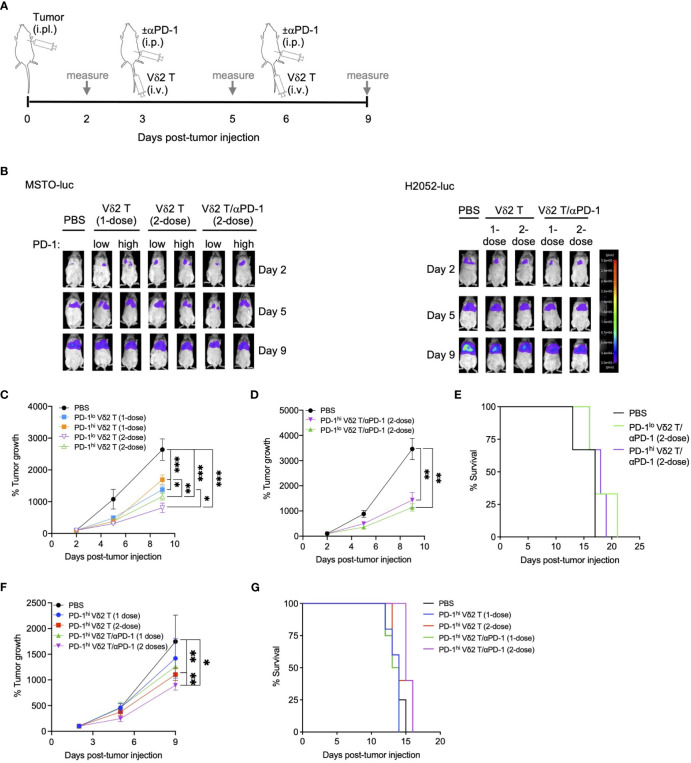
Vδ2 T cells and anti-PD-1 retard mesothelioma tumor growth *in vivo*. **(A)** Timeline of both MSTO-luc and H2052-luc mice experiment indicating the day for cell or αPD-1 injection, or measuring tumor size based on luciferase activity in tumor bearing mice. **(B)** Representative images of luciferase activities of MSTO-luc and H2052-luc bearing mice receiving PD-1^lo^ Vδ2 T with or without αPD-1 at 1-dose or 2-dose regimen. PBS injection served as control. **(C, D)** Tumor growth measured by luciferase activities over time compared to day 2 post MSTO-luc tumor injection for different treatment groups. Each group contains 3 to 6 mice. **(E)** Kaplan-Meier plot for MSTO-luc mice receiving 2-dose of PD-1^lo^ or PD-1^hi^ Vδ2 T with αPD-1 over time. **(F)** Tumor growth of H2052-luc mice receiving one or two doses of PD-1^hi^ Vδ2 T with or without αPD-1 over time, with survival as Kaplan-Meier plot **(G)**. Each group contains 4-5 mice. Data represents mean ± SEM. Two-way ANOVA statistical test was used for analyzing tumor growth curves. *P< 0.05, **P< 0.01, ***P< 0.001.

To test whether the combination of Vδ2 T cells and αPD-1 could be effective *in vivo*, we first established a mouse model of pleural mesothelioma. We injected NOD.Cg-*Prkdc^scid^Il2rg^tm1Wjl^*/SzJ (NSG) immunodeficient mice, aged 4-6 weeks, with 5 x 10^6^ MSTO-luc or H2052-luc cells intrapleurally (i.pl). *In vivo* imaging showed xenograft detection in the upper body as early as two days post-injection, with signals increasing over time (**Figures 2A–C**, **Supplementary Figures 3A**, **4A**). Tumor masses were observed in the pleural cavity, mesothelium, pleural lining, and pericardial lining (**Supplementary Figures 3C**, **4C**).

Having established this model, we aimed to test the effectiveness of Vδ2 T cells combined with or without αPD-1 in these MSTO-luc and H2052-luc bearing mice. To validate the *in vitro* cytotoxicity data, we injected PD-1^lo^ and PD-1^hi^ Vδ2 T cells into the MSTO-luc bearing mice on day 3 and/or day 6 post-tumor injection (**Figure 2A**). One dose of intravenous (i.v.) injection of PD-1^lo^ or PD-1^hi^ Vδ2 T cells reduced tumor growth by ~48% and ~36%, respectively, as measured by tumor luciferase activity (**Figures 2B, C**). However, two doses of PD-1^lo^ Vδ2 T cells further reduced tumor by another ~10%, whilst PD-1^hi^ Vδ2 T cells improved it by ~12% (**Figure 2C**). Next, we sought to determine the effect of intraperitoneal (i.p.) αPD-1 treatment in combination with a 2-dose Vδ2 T cell adoptive immunotherapy in MSTO-luc mice in a separate experiment (**Figure 2A**). As shown in **Figure 2D**, two doses of PD-1^hi^ Vδ2 T cells with αPD-1 resulted in ~58% reduction in tumor growth compared to PBS control. Two doses of PD-1^lo^ Vδ2 T cells with αPD-1 resulted in ~67% reduction in tumor growth significantly. Control mice with tumor survived for 17 days, but those that received the PD-1^lo^ or PD-1^hi^ Vδ2 T cells with αPD-1 treatments survived for up to 21 days (**Figure 2E**), despite the significant tumor regression.

In the H2052-luc tumor-bearing mice, we tested the effectiveness of αPD-1 treatment in improving PD-1^hi^ Vδ2 T cells adoptive immunotherapy (**Figure 2F**). Two doses of PD-1^hi^ Vδ2 T cells showed an advantage over one dose in reducing tumor growth, by ~37% and ~19%, respectively. Further, one and two doses of PD-1^hi^ Vδ2 T cells/αPD-1 were able to decrease tumor growth by ~28% and ~49%, respectively. These data suggest that immune checkpoint blockade for Vδ2 T cells with high PD-1 expression confers a better prognosis for decreasing mesothelioma tumor mass. However, survival was only prolonged for one day with two doses of combined treatment in 33% of mice compared to control (**Figure 2G**). For the mice experiments, no significant weight loss was detected (**Supplementary Figures 3B**, **4B**).

### Expression of Vδ2 T cell activating and inhibitory molecules in mesothelioma

BTN2A1/BTN3A1 are essential molecules for the activation of Vδ2 T cells (32), as we have previously shown their detection in solid tumors (21). As the results suggest that Vδ2 T cells have certain effectiveness against mesothelioma, we next examined the expression of BTN2A1, BTN3A1, and PD-L1 in mesothelioma tumor tissue sections obtained from the mice experiments described earlier, at the time of death between 17-21 days.

**Figure 3:**
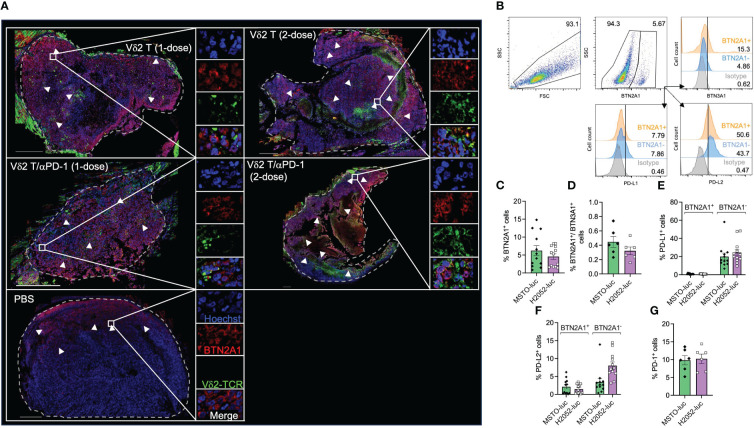
Analysis of Vδ2 T cell infiltration to BTN2A1^+^ mesothelioma cells, and expression of BTN2A1, BTN3A1, PD-L1 and PD-L2. **(A)** H2052-luc tumor tissue sections from PBS, Vδ2 T (1-dose), Vδ2 T (2-dose), Vδ2 T/αPD-1 (1-dose), and Vδ2 T/αPD-1 (2-dose) mice were immunostained for Vδ2-TCR^+^ (green), BTN2A1 (red), and nucleus (blue) with Hoechst 33258. Scale bar represents 500 μm. Representative tile scan images acquired by confocal microscopy are shown. Insets are expanded views of the rectangular regions. Dashed lines indicate the perimeter of the tumor. White arrows indicate regions of BTN2A1-expressing cells. **(B–G)** Flow cytometric analysis of the expression of BTN2A1, BTN3A1, PD-1, PD-L1, PD-L2 on MSTO-luc and H2052-luc cells. Gating strategy is shown in **(B)** with analysis for the expression (numbers are percentages) shown as column graphs for **(C)** BTN2A1^+^, **(D)** BTN2A1^+^/BTN3A1^+^, **(E)** PD-L1^+^, **(F)** PD-L2^+^ and **(G)** PD-1^+^ on MSTO-luc and H2052-luc cells. Data represents mean SEM from ≥ 6 independent experiments.

As shown in **Supplementary Figure 5A**, BTN2A1, BTN3A1, and PD-L1 appears to express differentially on cells and in different regions by immunofluorescence and tile scan from confocal microscopy. Vδ2-TCR^+^ cells were observed to have infiltrated into the tumor, with the cells present in 14% of the tumor area for the two-dose treatment, compared to 9% for the one-dose treatment (**Figure 3A**). Moreover, Vδ2-TCR^+^ cells seemed to be located in regions where tumor cells expressed BTN2A1 (**Figure 3A**) and were found to coincide with cells that co-expressed PD-L1 (**Supplementary Figure 5A**). However, there are also regions in the tumor that only expressed BTN2A1, BTN3A1/PD-L1 or BTN2A1/BTN3A1/PD-L1 (**Supplementary Figure 5A**). In the tumors of mice that also received αPD-1 treatment along with Vδ2 T cells, Vδ2 T cells tended to localize to BTN2A1^+^ cells, similar to those mice that received Vδ2 T cells only (**Figure 3A**). To further illustrate the characteristics of the tumor cells in the microenvironment, we quantified the cells expressing combinations of BTN2A1, PD-L1, and/or PD-L2 in the tile scan of the tumor section from the two-dose treatment (**Supplementary 5B**). Out of the 24,636 nucleated cells counted, 29.7% did not express these markers, while 26.8% expressed all three markers. Interestingly, cells expressing BTN2A1 or BTN3A1 alone accounted for 27.5% and 2.0% of cells, respectively. 5.9% of cells co-expressed BTN2A1/BTN3A1, suggesting that Vδ2 T cells with full functional capacity could potentially target 32.7% of cells in the tumor if unhindered by the presence of PD-L1 or blocked by αPD-1. By flow cytometry, we analyzed MSTO-luc and H2052-luc cell lines for the expression of BTN2A1, BTN3A1, PD-L1 and PD-L2 (**Figure 3B**), BTN2A1 expression accounted for ~6.2% of MSTO-luc and ~4.6% of H2052-luc cells (**Figure 3C**). However, only<1% of cells co-expressed surface BTN2A1 and BTN3A1 (**Figure 3D**). A higher frequency of cells with PD-L1 expression was found in BTN2A1^-^ (MSTO-luc: ~20%, H2052-luc: ~20%) compared to BTN2A1^+^ (MSTO-luc: ~0.91%, H2052-luc: ~0.79%) sub-populations (**Figure 3E**). PD-L2 expression was found on ~2.1% of BTN2A1^+^ and ~3.4% of BTN2A1^-^ cells for MSTO-luc, and ~1.5% of BTN2A1^+^ and ~8.0% of BTN2A1^-^ cells for H2052-luc (**Figure 3F**). The expression of these molecules may explain the difference in the susceptibility of the cell lines towards Vδ2 T cell cytotoxicity (**Figure 1**). Moreover, we considered the expression of PD-1 on the mesothelioma cells (33), but it was found in only ~10% of cells (**Figure 3G**), and the expanded Vδ2 T cells exhibited an average 1.7±2.2% PD-L1 expression. Therefore, our analysis reveals that the expression of BTN2A1/BTN3A1 is low on the tumor cells in mesothelioma, where a high percentage of cells express PD-L1/PD-L2, which could hinder the effectiveness of Vδ2 T cells for immunotherapy. This suggests the necessity of αPD-1 co-treatment.

### Vδ2 T cells induce pyroptotic cell death in mesothelioma cells

**Figure 4:**
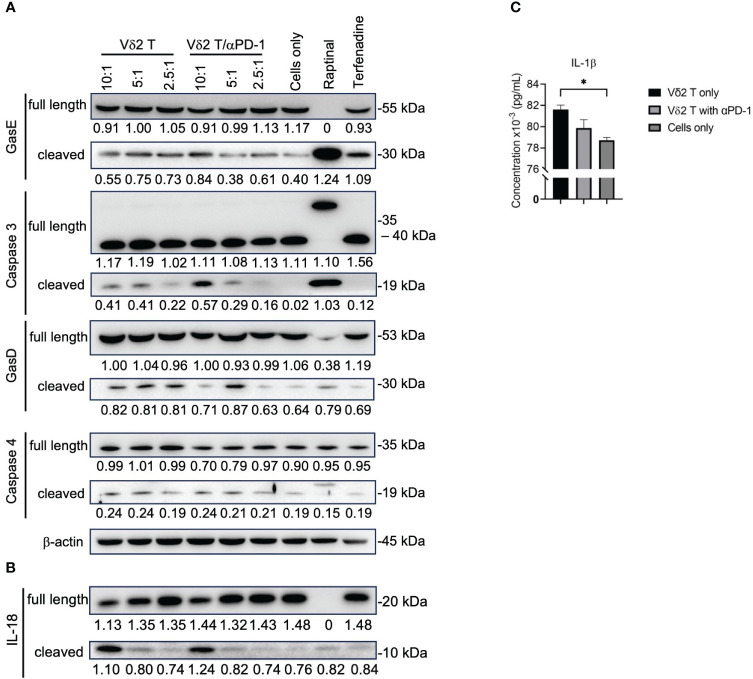
Vδ2 T cells induce pyroptosis in mesothelioma cells. **(A)** Analysis of protein expression level of gasdermin (Gas)E, caspase 3, GasD, caspase 4, and **(B)** IL-18, following 6 h of co-culture between Vδ2 T cells and MSTO-luc. Controls include raptinal and terfenadine, or cells only. Arrows indicate expected band size of the full-length or cleaved proteins. Numbers under the bands represent band intensity normalized to β-actin. Representative immunoblots are shown. **(C)** ELISA analysis of IL-1β release following 6 h of co-culture between Vδ2 T cells and MSTO-luc at 10:1 ratio. plotted as a column graph of mean ± SEM. Data from three independent experiments are shown. One-way ANOVA statistical test was used. *P< 0.05.

By performing live-imaging of the co-culture between Vδ2 T cells and mesothelioma cells, we observed the membrane blebbing (or “ballooning”) phenotype while the tumor cells were undergoing cell death (**Supplementary Video 1** and **Supplementary Figure 6**), which is indicative of pyroptosis. To confirm whether Vδ2 T cells are capable of inducing pyroptosis, western blot was used to determine whether the cleavage forms of gasdermin D (GasD), gasdermin E (GasE), caspase 3, 4 could be induced by co-culture of Vδ2 T cells with MSTO-luc cells. Tumor cells only served as negative control. Raptinal and terfenadine treatments served as positive controls for caspase 3 and caspase 4 activation, respectively. After 6 h of co-culture, there was increased level of cleaved caspase 3, particularly at the 10:1 E:T ratio (**Figure 4A**). αPD-1 treatment resulted in higher level of cleaved caspase 3 for the 10:1 ratio, but to a lesser extent with 5:1 and 2.5:1 ratios (**Figure 4A**). While cleavage of GasE (which can be mediated by caspase 3) seems to have a corresponding effect at 10:1 (**Figure 4A**). Band intensity values and the ratio of cleaved GasE over full-length GasE, and other proteins, are shown in **Supplementary Figure 7**. There was a small increase in cleaved GasD compared to control, particularly at the 5:1 ratios for Vδ2 T and/or αPD-1 (**Figure 4A**). However, cleaved caspase 4, and full-length forms of caspase 3, caspase 4, GasD and GasE were similar between the treatments (**Figure 4A**), suggesting that co-culture of Vδ2 T and MSTO-luc induced the active form of GasE likely due to caspase 3, and active form of GasD not due to caspase 4. While raptinal induced clear caspase 3 and GasE activation, terfenadine was not successful for caspase 4 and GasD in the mesothelioma cell lines (**Figure 4A**). In contrast, the effect on the active form of IL-18 was more obvious at 10:1 ratio co-culture, with a coupled decrease of pro-IL-18 (**Figure 4B**). However, αPD-1 did not increase the level of active IL-18 detected even at the 10:1 ratio. In concordance, there was an increased level of IL-1β released at 10:1 ratio compared to target cells alone, but addition of αPD-1 treatment had no improvement (**Figure 4C**). For H2052-luc co-cultured with Vδ2, cleavage of caspase 3, GasE and IL-18 can be found only at the 10:1 ratio but not for GasD, caspase 4 or IL-1β (**Supplementary Figures 8A, B**). Interestingly, Vδ2 T cells did not induce cleaved form of caspase 3 in nasopharyngeal carcinoma (NPC) cell lines (**Supplementary Figure 9**). Taken together, Vδ2 T cells can induce pyroptosis in mesothelioma cells via the canonical pathway but only induce active IL-18 and IL-1β in MSTO-luc but not H2052-luc cells, which may suggest the higher resistance of H2052-luc cells towards Vδ2 T cell killing.

**Figure 5:**
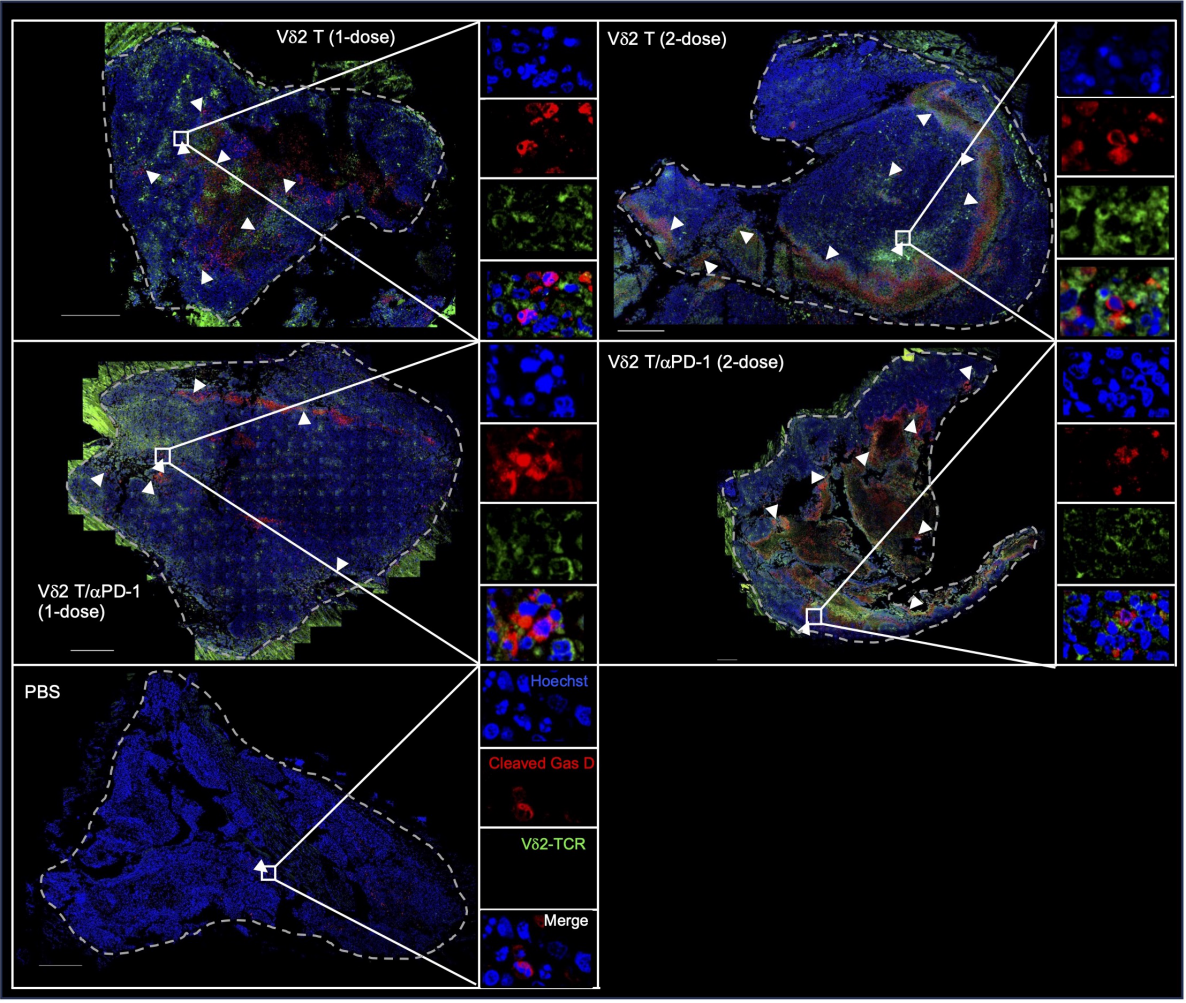
Vδ2 T cells induce pyroptosis in mesothelioma bearing mice. H2052-luc tumour tissue sections from PBS, Vδ2 T (1-dose), Vδ2 T (2-dose), Vδ2 T/αPD-1 (1-dose), and Vδ2 T/αPD-1 (2-dose) mice were immunostained for Vδ2-TCR (green), cleaved GasD (red), and nucleus (blue) with Hoechst 33258. Scale bar represents 500 μm. Representative tile scan images are shown. Insets are expanded views of the rectangular regions. Arrows indicate regions of cells with cleaved GasD expression.

To verify this *in vivo*, we examined H2052-luc tumor tissue sections from control mice or mice that received one or two doses of PD-1^hi^ Vδ2 T cell and/or αPD-1 treatment, for the expression of Vδ2-TCR and cleaved GasD from different treatment groups. Indeed, higher frequency of cleaved GasD was found adjacent to Vδ2-TCR^+^ cells in those that received two-dose treatment (19%) compared to one-dose treatment (8%) (**Figure 5**). However, we did not find noticeable difference for one or two doses of Vδ2 T cell treatment with αPD-1 to without αPD-1 (**Figure 5**). These data may suggest that the greater effect of tumor regression could be related to the level of pyroptosis induced by Vδ2 T cells.

## Discussion

Mesothelioma, which commonly affects the pleural cavity, poses a challenge for treatment due to its widespread nature and late-stage diagnosis. While immune checkpoint inhibitors have shown limited efficacy in treating this cancer (25, 26, 34), another promising approach is the use of chimeric antigen receptor (CAR)-T cell therapy, which has demonstrated high effectiveness against leukemia and certain solid tumors like neuroblastoma (35, 36). However, the lack of well-defined antigens specific to mesothelioma poses a significant obstacle in successfully implementing CAR-T cell therapy for this cancer. Nevertheless, clinical trials combining αPD-1 antibodies with CAR-T cells have shown promise, with median overall survival of mesothelioma patients potentially extended to around 20 months (37).

Alternatively, gamma-delta T cells have garnered attention as a non-MHC-restricted cell therapy for cancer due to their inherent tumor-killing and tumor-migratory properties (38). In the context of mesothelioma, a recent study demonstrated that Vδ2 T cells, when stimulated by PTA, exhibit high cytotoxicity against tumor cells, particularly at a ratio of 200:1 (23). Although this high ratio may raise clinical concerns, it provides valuable insight into the potential of Vδ2 T cells for combating this type of cancer. The study further revealed that these cells can engage three different killing mechanisms: NKG2D, T cell receptor (TCR), and CD16/antibody-dependent cell cytotoxicity (ADCC) (23). The practicality of testing the possible *in vivo* effects is realized in this study, where we utilized an immunodeficient NSG mouse model of pleural mesothelioma, enabling the adoptive transfer of human Vδ2 T cells. To the best of our knowledge, this is the first study to employ such a model for studying mesothelioma and evaluating human immune cell therapy. Indeed, using this model, we found that Vδ2 T cells can retard tumor growth. However, complete tumor elimination may require a larger number of Vδ2 T cells, which needs to be carefully considered due to the potential physiological burden associated with transferring up to nearly 200 times more cells than the tumor itself.

One may consider achieving high efficacy for mesothelioma by combining Vδ2 T cells with immune checkpoint inhibitor, such as anti-PD-1 antibody, to maintain the tumoricidal functions. Our work here demonstrated that PTA-expanded Vδ2 T cells from different donors exhibited varying levels of PD-1 expression, ranging from 0.5% to 30.9% of the cells. Notably, we found that anti-PD-1 treatment enhanced PD-1^hi^ Vδ2 T cells by at least ~10-20%, based on data from tumor size and cytotoxicity assay. This suggests that further refinement and development of this combination therapy are warranted, and would be necessary to conduct profiling of different clinical mesothelioma to better understand the tumor immune landscape. In this consideration, besides anti-PD-1, other immune checkpoint inhibitors such as anti-BTLA, anti-CTLA-4, anti-Siglec-10, anti-TIGIT, anti-TIM-3, anti-LAG-3 could be tested in combination to investigate their suitability for enhancing Vδ2 T cell efficacy in this mesothelioma model. Previous studies have identified that BTLA can suppress the proliferation of Vδ2 T cells (39), while blocking the PD-1 and CTLA-4 pathways can restore T cell function and improve survival in melanoma, colon carcinoma, and ovarian carcinoma (40, 41). Inhibition of Siglec-10 has been shown to decrease the expression of immune inhibitory molecules within the tumor microenvironment and enhance the anti-tumor activity of cytotoxic T lymphocytes in hepatocellular carcinoma (42). Simultaneous blockade of TIGIT and PD-L1 has elicited tumor rejection and antigen-specific protection in CT-26 tumor-bearing mice (43), and TIM-3 has been implicated in inhibiting the cytotoxic function of Vδ2 T cells by suppressing the release of granzyme B and perforin (44). Moreover, LAG-3 and PD-1 have been found to synergistically promote tumoral immune escape in melanoma and colon adenocarcinoma (45). Therefore, targeting these immune checkpoint molecules could help ensure the optimal function of Vδ2 T cells *in vivo*.

The induction of pyroptosis in mesothelioma cells by Vδ2 T cells is surprising. Pyroptosis is a type of inflammatory cell death that is characterized by the onset of inflammasome leading to the activation of caspase 3. Subsequently, cleaved caspase 3 activates GasE proteins, which migrate to the cell membrane, forming pores that allow osmosis and the formation of membrane blebbing due to cell swelling, ultimately resulting in cell rupture (10, 12). Simultaneously, the activated inflammasome caspase 1 cleaves pro-IL-18 and pro-IL-1β, releasing their active forms outside the cell through the GasE pore (10, 12, 14, 16). Alternatively, the non-canonical pathway can also lead to the formation of membrane pores through the activation of caspase 4, 5, or 11, which cleave GasD. In our study, we observed that co-culture of Vδ2 T cells with MSTO cells induced pyroptosis, as evidenced by the expression of caspase 3, GasE, GasD, IL-18, and IL-1β, which was confirmed through live-imaging. However, we did not observe stimulation of caspase 4 cleavage by Vδ2 T cells, suggesting that the cleaved GasD in MSTO cells may be mediated by caspase 5, caspase 8, or other mechanisms. While activation of pyroptosis has been demonstrated using various small molecules, chemotherapy drugs, or cell death inducers, this is the first instance where cell-cell interaction leading to pyroptosis has been shown for innate immune cells, particularly Vδ2 T cells. It would be worthwhile to investigate the cell surface proteins responsible for activating pyroptosis, as this knowledge could provide insights into enhancing the effectiveness of immune cell therapy by incorporating such proteins into the arsenal for combating cancer cells.

## Data availability statement

The original contributions presented in the study are included in the article/**Supplementary Material**. Further inquiries can be directed to the corresponding author.

## Ethics statement

The animal study was approved by (REC/22-23/0438) Animal Ethics Committee Hong Kong Baptist University. The study was conducted in accordance with the local legislation and institutional requirements.

## Author contributions

KL: Data curation, Formal Analysis, Investigation, Methodology, Writing – original draft, Writing – review & editing. ZY: Investigation, Methodology, Resources. HC: Investigation, Methodology. YT: Writing – review & editing, Resources. AC: Conceptualization, Data curation, Funding acquisition, Investigation, Methodology, Project administration, Supervision, Visualization, Writing – original draft, Writing – review & editing.
