# Supplementary material for: Anti-PD1 does not improve pyroptosis induced by γδ T cells but promotes tumor regression in a pleural mesothelioma mouse model
Source: Front Immunol. 2023 Nov 23;14:1282710. doi: 10.3389/fimmu.2023.1282710 (PMC10701743; doi:10.3389/fimmu.2023.1282710)
Supplement: Supplementary file 1 [file DataSheet_1.pdf]

Suppl. Fig. 1

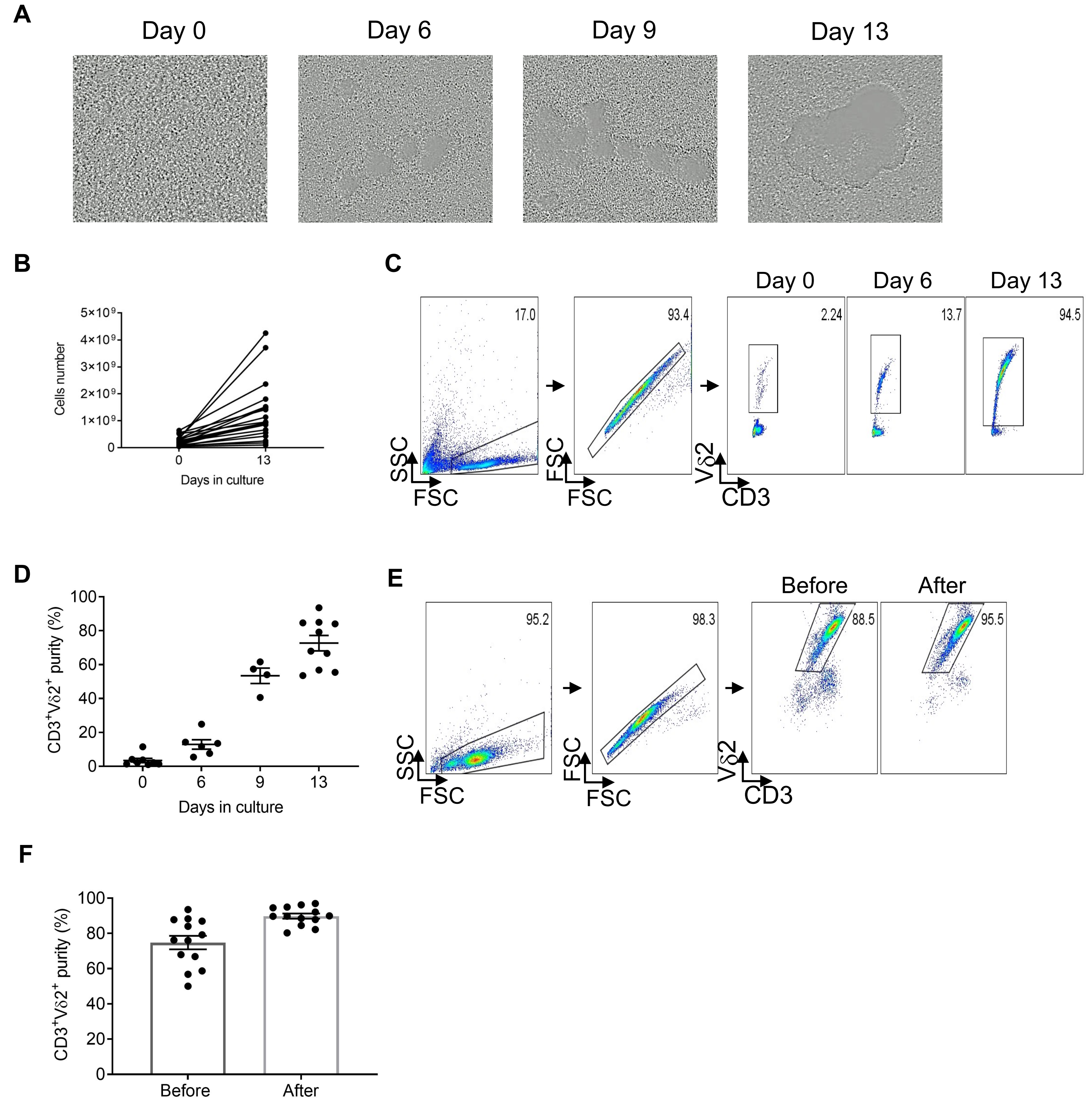

Suppl. Fig. 1. Expansion of Vδ2 T cells in vitro. (A) Detection of cell clusters observed under 40X bright field microscopy over time. **(B)** The number of $CD3^+V\delta 2^+$ cells increase ~100-fold after 13 days of culture (n=22). **(C, D)** Flow cytometry analysis of the purity of $CD3^+V\delta 2^+$ cells increased over time in the expansion culture. Representative dot plots and a column graph are shown (n≥4). **(E, F)** Dot plots shown for the purity of $CD3^+V\delta 2^+$ cells achieved to >95% after microbeads isolation presented as column graph (n=13).

Suppl. Fig. 2

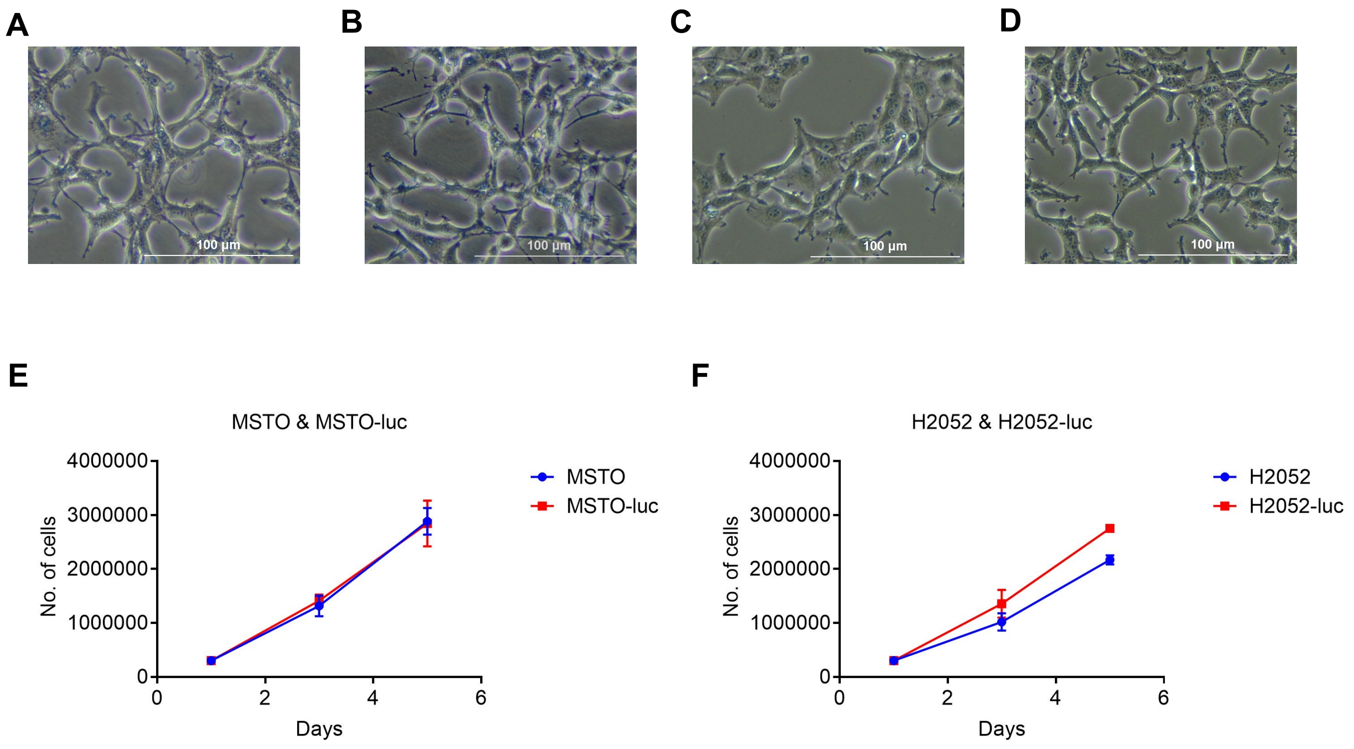

Suppl. Fig. 2. Cell growth rate of mesothelioma and transduced luciferase reporter cell lines. Morphology of **(A)** MSTO, **(B)** H2052, **(C)** MSTO-luc and **(D)** H2052-luc cell lines under bright field microscope at 100X magnification. Number of cells over time of **(E)** MSTO and MSTO-luc, and **(F)** H2052 and H2052-luc shown as line graphs.

Suppl. Fig. 3

A

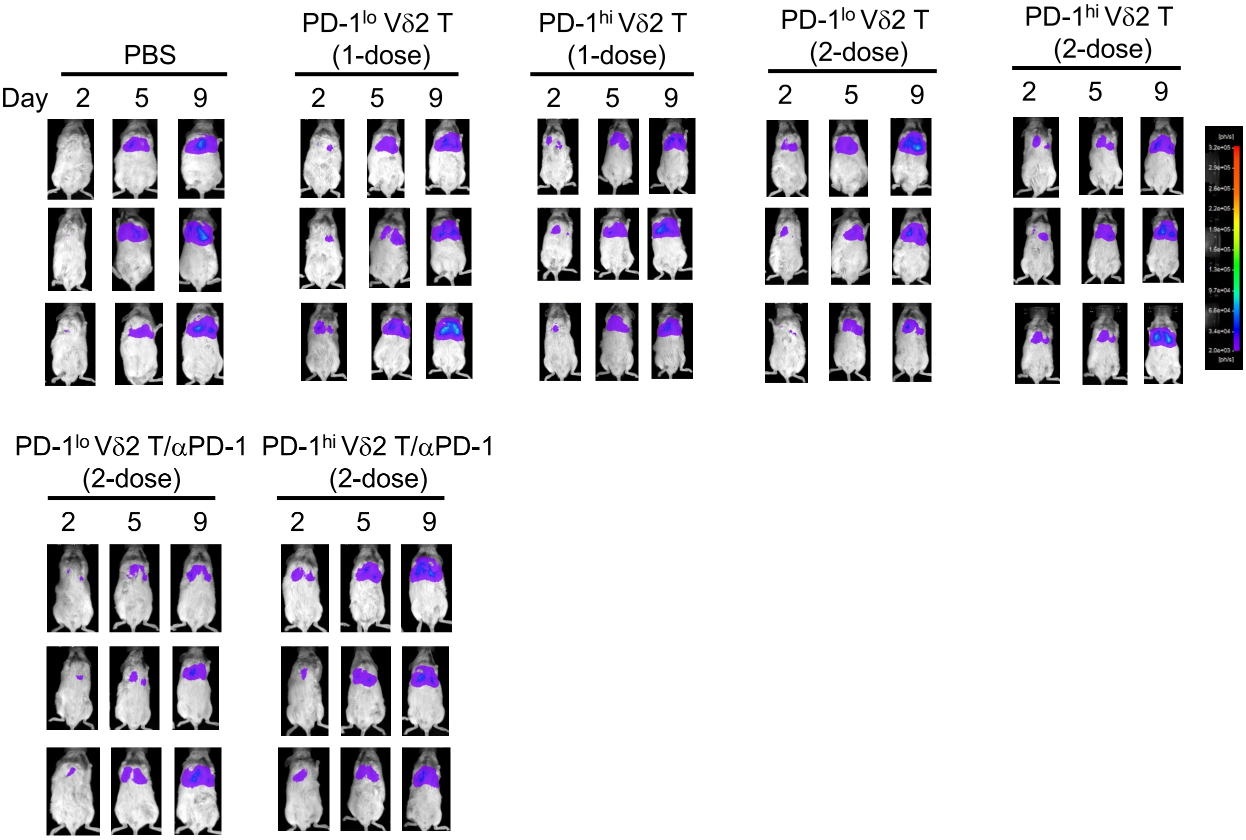

B

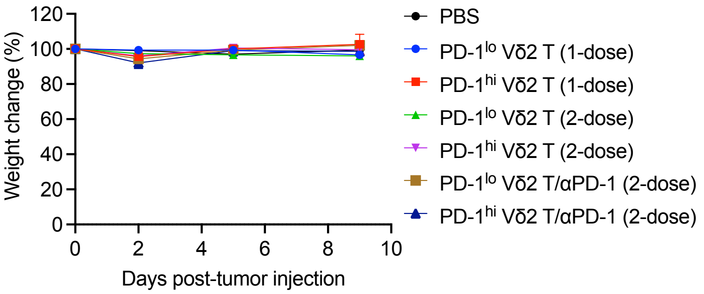

C

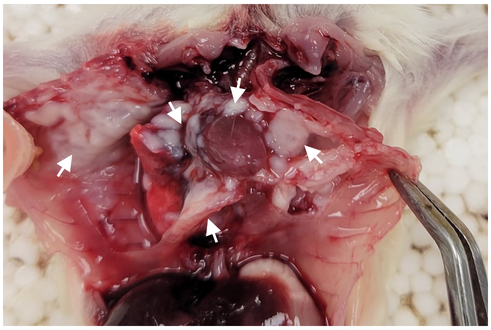

Suppl. Fig. 3. MSTO-luc mice experiments. (A) Detection of luciferase activities of MSTO-luc mice from each treatment group over the course of experiment. Three mice per group is shown. **(B)** Weight change of MSTO-luc mice groups. **(C)** Spread of MSTO-luc tumor at experimental endpoint in the pleural cavity, with white arrows indicating the tumor mass on the mesothelium, pleural and pericardial lining.

Suppl. Fig. 4

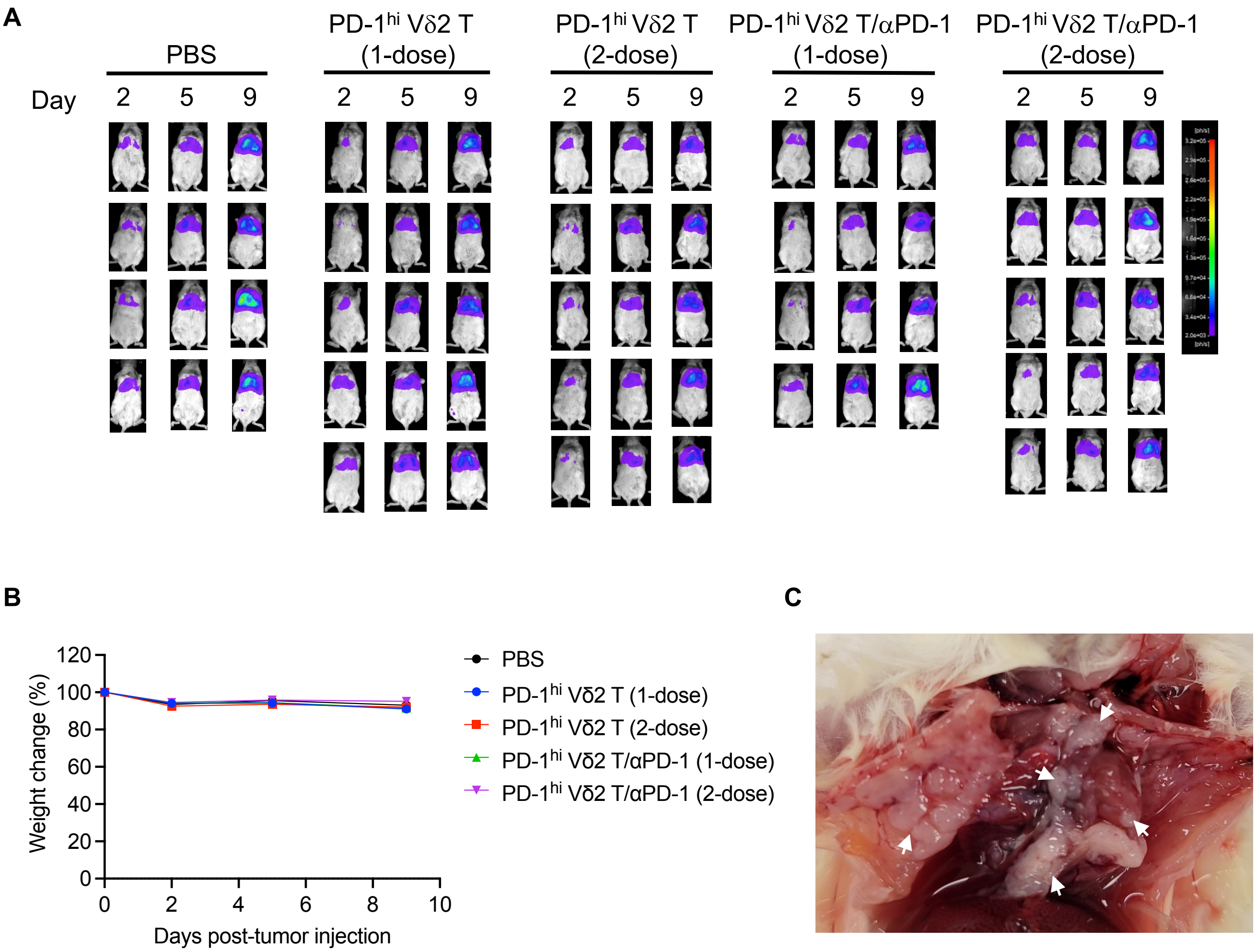

Suppl. Fig. 4. H2052-luc mice experiments. (A) Detection of luciferase activities of H2052-luc mice from each treatment group over the course of experiment. Four to five mice per group is shown. **(B)** Weight change of H2052-luc mice groups. **(C)** Spread of H2052-luc tumor at experimental endpoint in the pleural cavity, with white arrows indicating the tumor mass on the mesothelium, pleural and pericardial lining.

A **Suppl. Fig. 5**

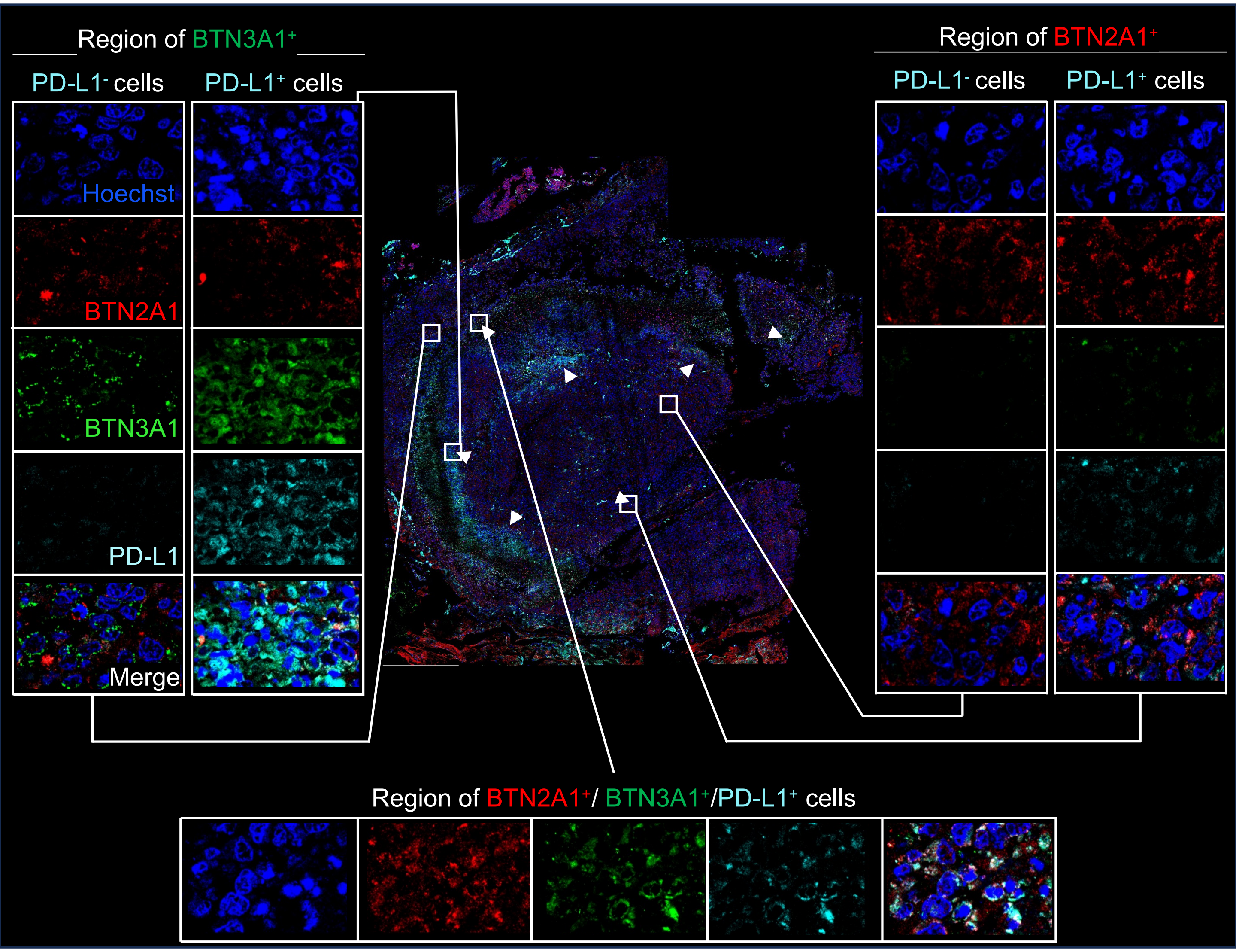

B

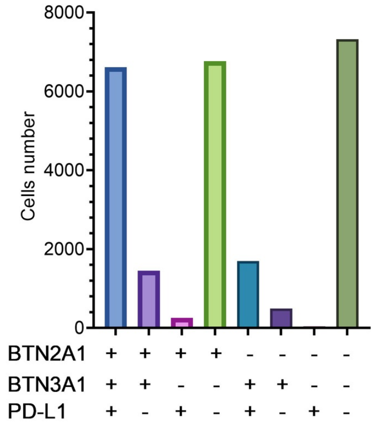

Suppl. Fig. 5. Analysis of BTN2A1, BTN3A1 and PD-L1 expressing cells in mesothelioma tumor. (**A**) H2052-luc tumor tissue sections from Vδ2 T (2-dose) mice were immunostained for BTN3A1 (green), BTN2A1 (red), PD-L1 (cyan) and nucleus (blue) with Hoechst 33258. Scale bar represents 500 μm. Representative tile scan images are shown. Insets are expanded views of the rectangular regions showing cells that are $BTN2A1^+/PD\text{-}L1^-$, $BTN2A1^+/PD\text{-}L1^+$, $BTN3A1^+/PD\text{-}L1^-$, $BTN3A1^+/PD\text{-}L1^+$, and $BTN2A1^+/BTN3A1^+/PD\text{-}L1^+$. Arrows indicate regions of PD-L1 expression. (**B**) Cell counts of different combinations of single, dual or triple positive cells in the tile scan are shown.

Suppl. Fig. 6

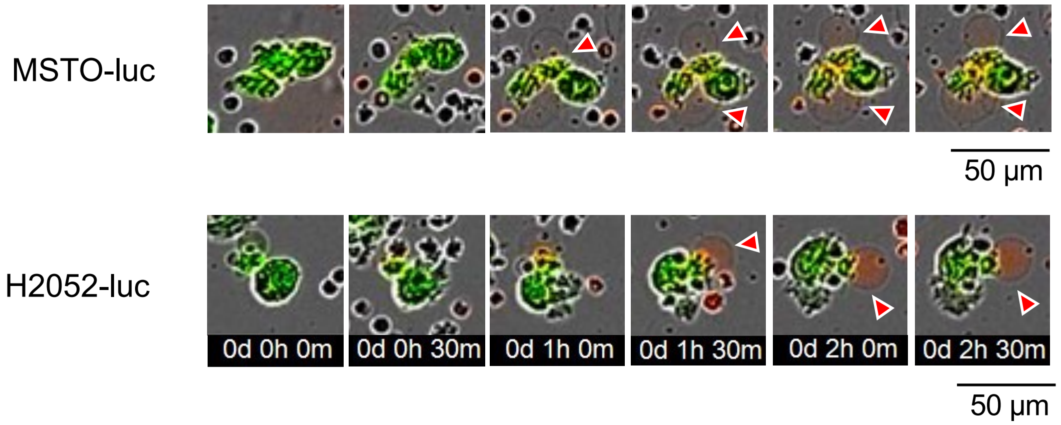

Suppl. Fig. 6. Membrane swelling of mesothelioma cells induced by co-culture of Vδ2 T cells at 10:1 E:T ratio. MSTO-luc and H2052-luc cells were pre-stained with green fluorescent Calcein dye. Cytotox Red reagent were used in the media, accumulation of red fluorescence indicates cell membrane integrity disruption (cell death). Arrows point to the membrane swelling or "ballooning" of the cells, a characteristic of pyroptosis.

Suppl. Fig. 7

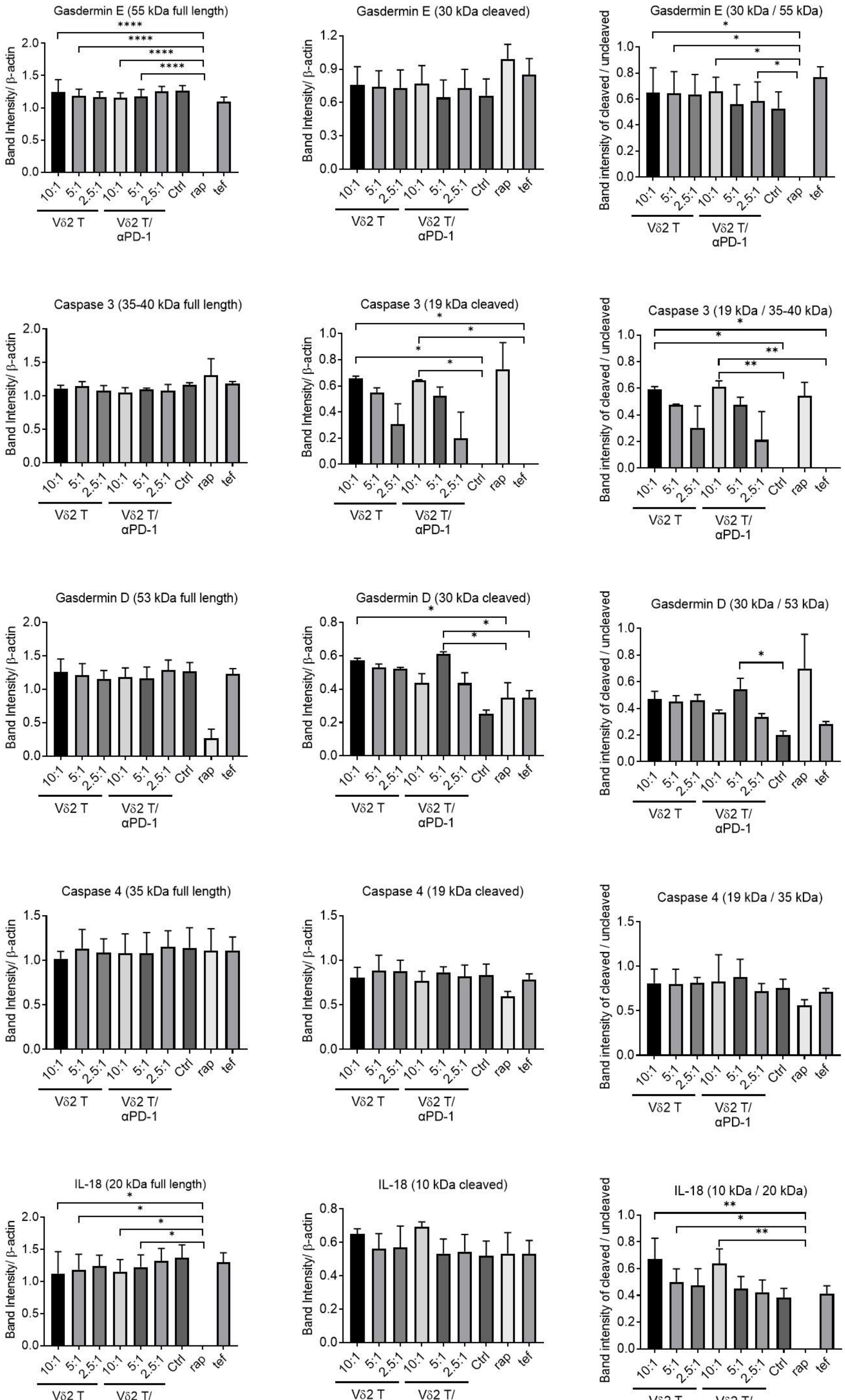

Suppl. Fig. 7. Densitometry analysis of pyroptosis related proteins from Western blotting bands. Band intensities normalized to β-actin from Figure 4 and two other independent experiments are shown as mean ± SEM in the column graphs. Ratio of cleaved and full-length forms of GasD, GasE, Caspase-3 and Caspase-4 are shown. One-way ANOVA statistical test was used. *$P < 0.05$, **$P < 0.01$, ****$P < 0.001$.

Suppl. Fig. 8

A

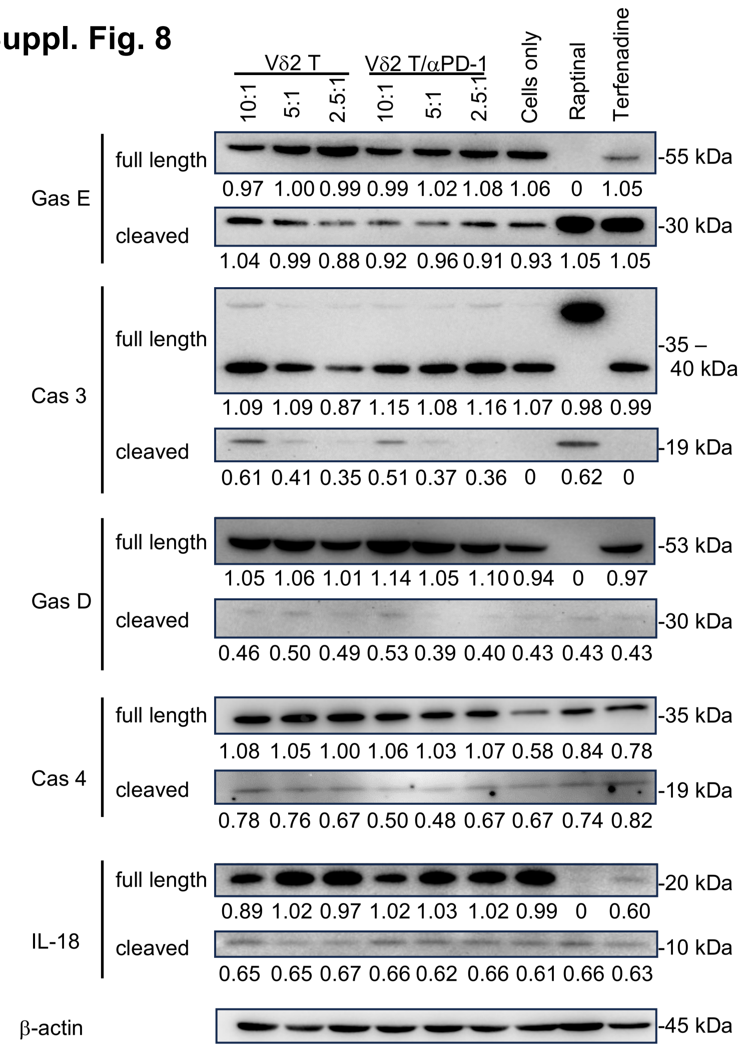

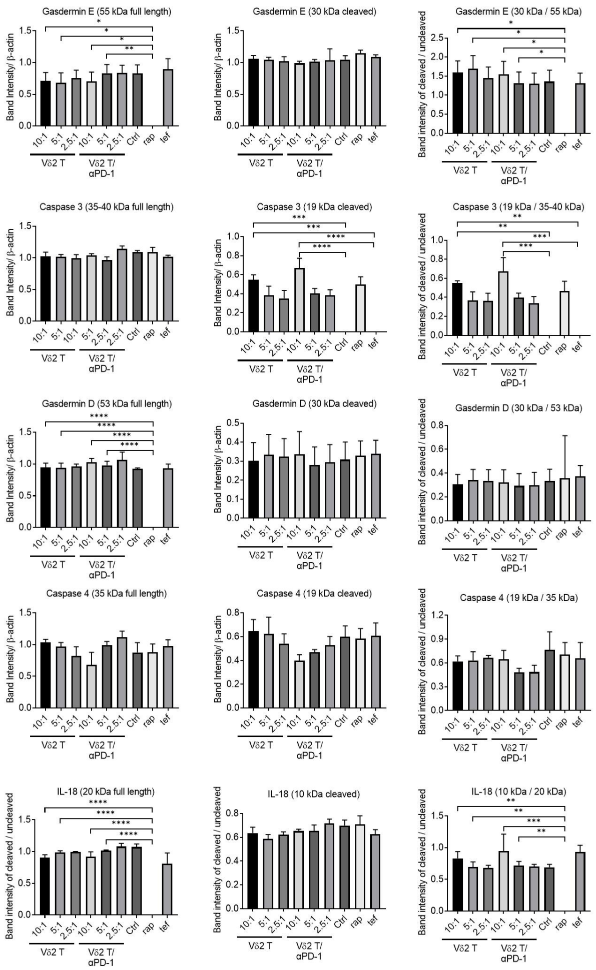

B

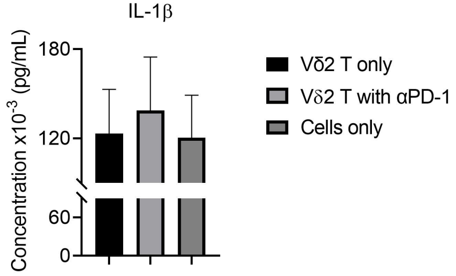

Suppl. Fig. 8. Vδ2 T cells elicit the expression of caspase 3, caspase 4, GasD, GasE and IL-18 in H2052-luc. (**A**) Analysis of protein expression level of GasE, caspase 3, GasD, caspase 4, and (**B**) IL-18, following 6 h of co-culture between Vδ2 T cells and H2052-luc. Arrows indicate expected band size of the full-length or cleaved proteins. Numbers under the bands represent band intensity normalized to β-actin. Representative immunoblots are shown. (**C**) ELISA analysis of IL-1β in H2052-luc in 10:1 effector to target ratio. Data represents mean ± SEM from three independent experiments. One-way ANOVA statistical test was used. *$P < 0.05$, **$P < 0.01$, ****$P < 0.001$.

Suppl. Fig. 9

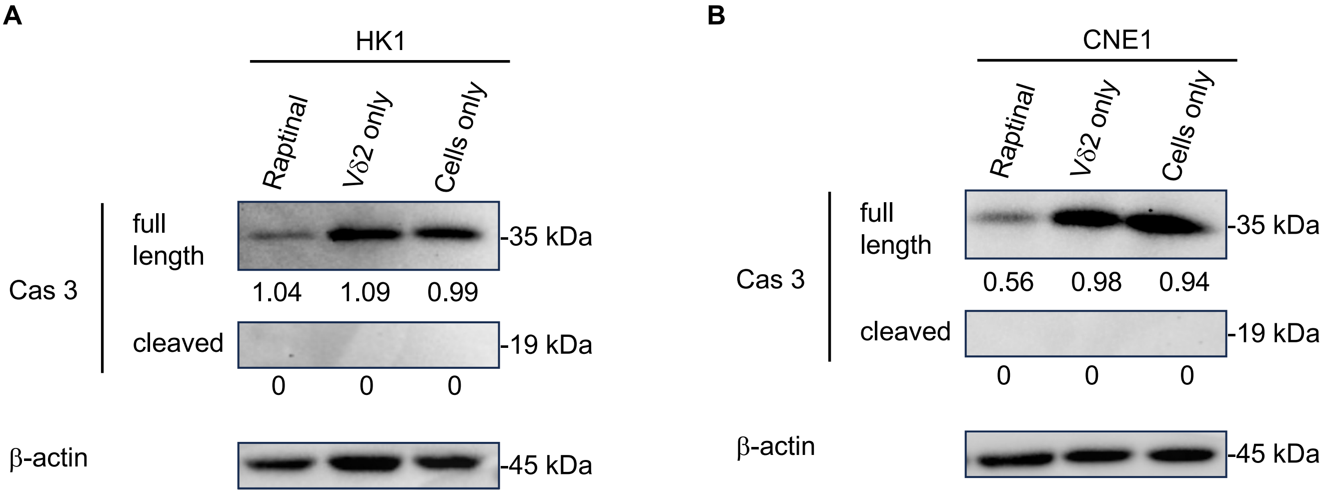

Suppl. Fig. 9. Expression of caspase 3 in HK1 and CNE1 following co-culture with Vδ2 T cells. Analysis of band intensities of Western blot compared to β-actin (shown as numbers) in **(A)** HK1, and **(B)** CNE1. Representative immunoblots are shown.

Suppl. Table 1. Antibodies used in this study.

Experiment	Antibody name	Host	Catalog no.	Source
Western blot	anti-β actin	rabbit	4970	Cell signaling
	anti-Caspase 3	rabbit	9662	Cell Signaling
	anti-Caspase 4	rabbit	ab22687	Abcam
	anti-Gasdermin E	rabbit	19453	Cell Signaling
	anti-Gasdermin D	rabbit	97558	Cell Signaling
	anti-IL-18	rabbit	67775S	Cell Signaling
	anti-rabbit	donkey	AP182P	Merck
Flow cytometry	PE anti-BTN2A1	rabbit	orb494685	Biorbyt
	FITC anti-BTN3A1	rabbit	abx303219	Abbexa
	APC anti-human CD274 (PD-L1)	mouse	563741	BD Pharmingen
	BV421 anti-human CD273 (PD-L2)	mouse	563842	BD Horizon
	Alexa Fluor 700 anti-human CD95 (Fas)	mouse	305648	Biolegend
	PE anti-human Vδ2 TCR	mouse	555739	BD Pharmingen
	Pacific Blue anti-human CD3	mouse	558117	BD Pharmingen
	APC anti-human CD279 (PD-1)	mouse	558694	BD Pharmingen
Immunohistochemistry	anti-BTN3A1	mouse	ABIN5684130	Antibodies onlince.com
	anti-BTN2A1	rabbit	orb499606	Biorbyt
	TCR V delta 2 monoclonal antibody (15D)	mouse	TCR1732	Invitrogen
	CD274 (PD-L1, B7-H1) monoclonal antibody (MIH5)	rat	14-5982-82	Invitrogen
	Cleaved gasdermin D	rabbit	36425S	Cell Signaling
	Alexa Fluor 488 anti-mouse	goat	A-11005	Invitrogen
	Alexa Fluor Plus 647 anti-rabbit	goat	A32733	Invitrogen
	Alexa Fluor 594 anti-rat	goat	A-11007	Invitrogen
	Hoechst 33258		ab228550	Abcam
